# Malignant transformation of a maxillary follicular ameloblastoma to squamous cell carcinoma: A case report and review of literature

**DOI:** 10.1016/j.ijscr.2025.111560

**Published:** 2025-06-23

**Authors:** Hanae Ben Abdenbi, Hafsa El Ouazzani, Habiba Kadiri, Firdaous Touarsa, Nadia Cherradi

**Affiliations:** a Department of Pathology HSR, Ibn Sina University Hospital Center, Faculty of Medicine and Pharmacy, Mohammed V University, Rabat, Morocco; b Department of Radiology HSR, Ibn Sina University Hospital Center, Faculty of Medicine and Pharmacy, Mohammed V University, Rabat, Morocco

**Keywords:** Squamous cell carcinoma, Ameloblastoma, Malignant transformation, Maxilla, Case report

## Abstract

**Introduction:**

Ameloblastoma is a benign but locally infiltrative epithelial odontogenic neoplasm of the jawbones. Ameloblastoma and squamous cell carcinoma commonly affect the mandible and maxilla. However, malignant transformation of ameloblastoma into squamous cell carcinoma is extremely rare.

**Case presentation:**

A 72-year-old man presented with a rapidly enlarging mass in the maxilla. Diagnostic imaging revealed a large mass with lytic changes involving the anterior and left aspects of the maxilla, and extending superiorly into the maxillary sinus. The patient underwent curettage treatment for a follicular ameloblastoma. The histological examination of the surgical resection specimen showed a close correlation between squamous cell carcinoma and ameloblastoma. On immunohistochemical analysis, staining for p53 and ki67 was positive in the squamous cells but not in ameloblastoma cells. The patient received subsequently radiation therapy with no evidence of recurrence 12 months post operatively.

**Discussion:**

Malignant transformation of ameloblastoma into squamous cell carcinoma is very rare, accounting for approximately 0.1 % to 1.8 % of all oral cancers. The first diagnostic challenge is to rule out the primary intraosseous squamous cell carcinoma using Gardner's criteria. In this case, the lesion was difficult to diagnose as a malignant transformation based only on intraoral examination and CT images. Histological and immunohistochemical analysis are gold standards for establishing the diagnosis.

**Conclusion:**

In summary, this report aims to discuss the possibility of malignant progression of follicular ameloblastoma into squamous cell carcinoma through a literature review. We are also targeting to highlight the essential rule of histological and immunohistochemical studies to establish the diagnosis.

## Highlights

• Malignant transformation of ameloblastoma into squamous cell carcinoma is extremely rare.
• Malignant transformation of an ameloblastoma may be suspected based on clinical and radiological findings, however, the diagnosis is made through histological and immunohistochemical examination of a representative biopsy sample.
• Early surgical resection followed by adjuvant radiotherapy remains the treatment of choice for avoiding recurrence.
• Close monitoring and careful follow-up are essential for the early detection of any signs of recurrence. Its frequency depends on each individual case.

## 1 Introduction

Ameloblastoma is the most common odontogenic benign tumor that stems from dental embryonic remnants in the mandible or maxilla. Several morphologic patterns exist including follicular, plexiform, and less frequently, acanthomatous, granular, basaloid, spindle cell and desmoplastic [1].

Malignant transformation of ameloblastoma to squamous cell carcinoma is extremely rare, and the pathogenesis is still controversial. The incidence of this tumor is similar in both sexes and is estimated to represent between 0.1 % and 1.8 % of all oral cancers [2]. Roughly, 80 % of these lesions affected the mandible with 2/3 in the posterior mandible. Whereas the maxilla was affected in 21 % of the cases [3].

The diagnosis is usually based on a combination of patient history, clinical examination, X-ray findings, and typical histologic characteristics of a representative biopsy sample. Once the diagnosis is established, patients typically undergo surgical resection with or without reconstruction, followed by radiotherapy.

This case report has been reported in line with the SCARE checklist, reporting the malignant transformation of a maxillary follicular ameloblastoma into squamous cell carcinoma in a 72-year-old man. A review of the literature was also carried out to clarify the criteria suggesting malignant degeneration [4].

## 2 Case report

A 72-year-old man presented to the Department of Maxillofacial Surgery with a 3-month history of a rapidly enlarging and painless mass in the left maxilla without dysphagia or epistaxis.

The patient reported arterial hypertension as the only pathological antecedent. He had never smoked or drank and no familial history of malignancy was noted.

Clinical examination showed an asymmetrical face and swelling towards the anterior and the left side of the upper jaw.

An oral examination revealed a submucosal, slightly elevated and indurated mass, measuring 9 cm in diameter, which involved the veil of the palate and extended to the left body of the maxilla. In the neck examination no lymphadenopathy was found and the remaining physical examination was unremarkable.

**Fig. 1:**
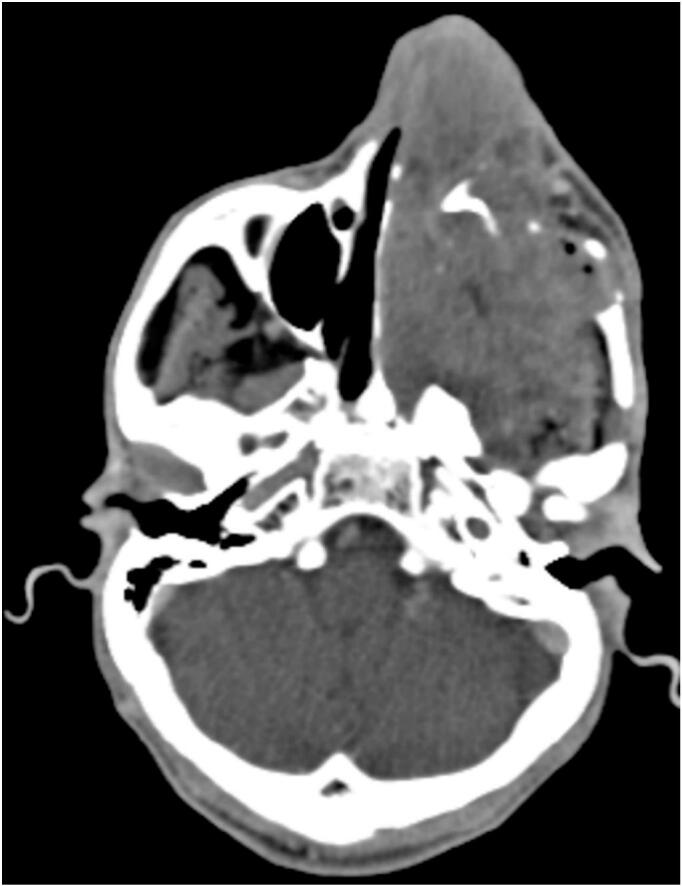
Maxillofacial CT scans showing an expansile radiolucent/radiopaque lesion, with lytic changes, located in the anterior left maxilla, extending superiorly into the left maxillary sinus.

Maxillofacial computed tomography (CT) demonstrated an expansile and radiolucent/radiopaque lesion, with lytic changes, located in the anterior maxilla, extending superiorly into the left maxillary sinus. The lesion exhibited low attenuation, irregular and poorly defined margins and an ovoid shape. Its largest dimension measured approximately 9 cm. Post-contrast imaging revealed heterogeneous enhancement, indicative of variable internal vascularity (Fig. 1).

## 3 Pathologic findings

Five biopsy specimens of the maxillary mass, measuring between 1.0 × 0.3 × 0.2 cm and 0.1 × 0.1 × 0.1 cm, were received and embedded in totality for microscopical examination.

**Fig. 2:**
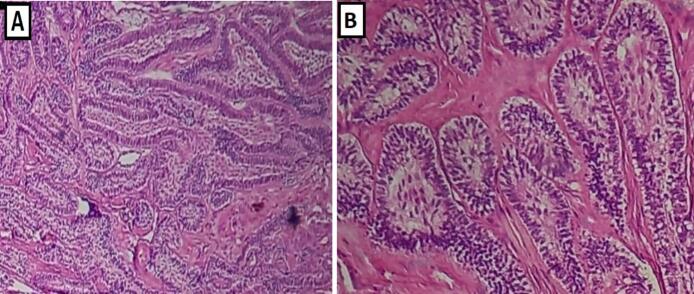
Histological aspects of biopsy specimen in Hematoxylin and Eosin staining (H&E). (A) H&E × 10; Low-power view showing benign component of the lesion with characteristic morphologic features of follicular ameloblastoma. (B) H&E × 20; Higher magnification showing follicular nests, composed of palisading peripheral cuboidal cells surround central stellate reticulum.

Histopathological study of step serial sections showed a benign odontogenic epithelial tumor, arranged in follicular nests, composed of palisading peripheral cuboidal-to-columnar basal cells. These cells have a vacuolated cytoplasm and hyperchromatic nuclei demonstrate a reversed polarity. The central epithelial areas were composed of polygonal cells suggestive of stellate reticulum. There were no areas of cellular atypia, mitotic figures or other signs of malignancy (Fig. 2).

The lesion was diagnosed as a follicular ameloblastoma. However, the rapid growth of the primary tumor and the aggressive clinical course suggested the possibility of malignancy.

Three months later, the patient underwent transoral surgical resection of the tumor and curettage for a follicular ameloblastoma without reconstruction. The tumor was received in several whitish fragments, measured between 1x1x0,9 and 9 × 7 × 5 cm, and weighed together 160 g. They were friable in consistency, with an irregular surface and partially covered by a mucosa.

Histologically, the surgical specimen revealed a biphasic neoplasm with both malignant and benign epithelial components in an extensive fibrous stroma.

Most of the tumor showed classic morphologic features of benign follicular ameloblastoma. It consisted of sheets of peripheral cuboidal ameloblastic cells that demonstrate reversed polarity and are arranged in a palisaded pattern, with an inner area of stellate reticulum.

The malignant epithelial component represents 20 % of the tumor surface, forming infiltrating nests and islands of moderately differentiated squamous cell carcinoma with low keratinization. The carcinoma cells showed nuclear pleomorphism with obvious nucleoli. Atypical mitotic activity was also present.

**Fig. 3:**
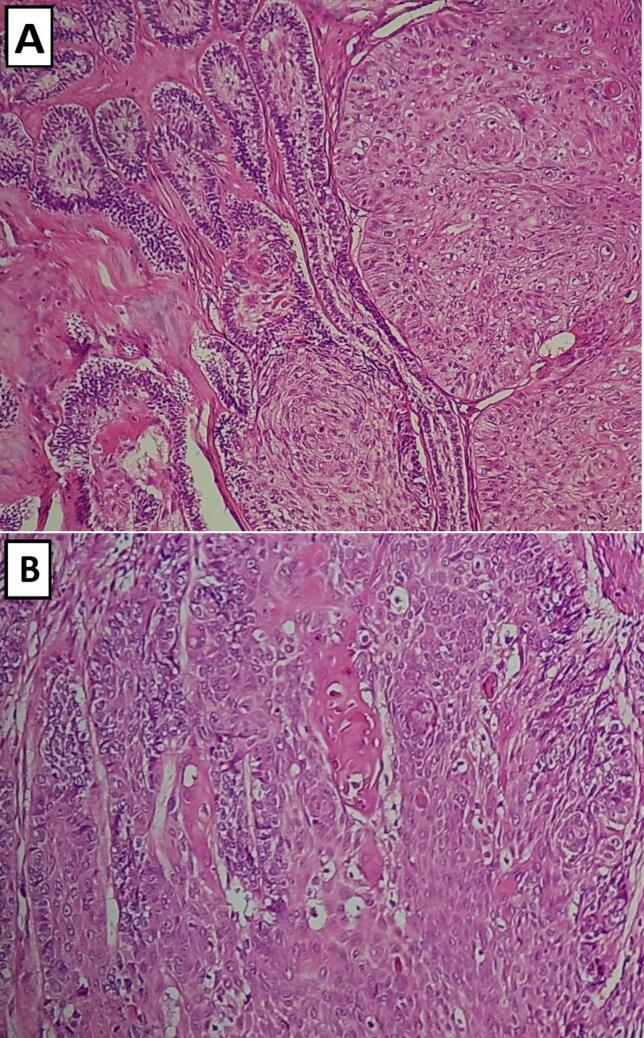
Sheets of moderately differentiated squamous cell carcinoma in juxtaposition with benign follicular ameloblastoma: (A) H&E × 20; (B) H&E × 40.

There was no morphological evidence suggesting mucosal origin of squamous cell carcinoma in the resected tumor. However, the areas of the benign odontogenic epithelium were closely located to the invasive nests and the overlapping of the two components was focally evident (Fig. 3).

P53 and Ki-67 immunohistochemical stains had the best performance to classify and characterize the tumor. They illustrated a distinct difference between the benign and malignant epithelial components.

**Fig. 4:**
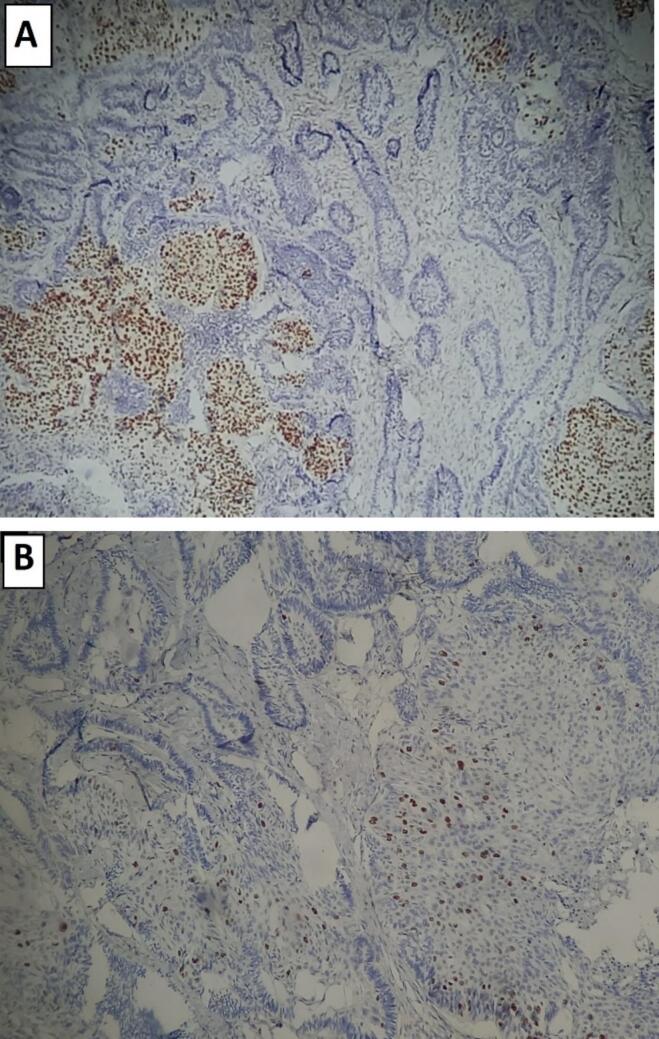
(A) p53 immunohistochemical stain showing lower nuclear expression in the benign ameloblastic component (right area) and higher nuclear expression in the malignant squamous cell carcinoma component (left area), (B) Ki-67 immunohistochemical stain showing differences between the proliferation indices of the benign (left and higher area) and malignant epithelial components (right and low area).

The tumor cells were positive for p53 with stronger and wider staining within the malignant component than in the follicular ameloblastoma (Fig. 4A). Likewise, Ki-67 showed diffuse positivity in the malignant nests estimated at around 30 %, compared to 1 % in the ameloblastoma (Fig. 4B).

There was no vascular invasion, no peri-nerve sheath involvement, or metastatic lymph nodes; however, the surgical margins were negative.

According to the histological and immunohistochemical findings, the final diagnosis was moderately differentiated squamous cell carcinoma arising from follicular ameloblastoma of the maxilla.

The patient refused left total maxillectomy and titanium plate reconstruction. Subsequently, he underwent a course of radiotherapy without concurrent systemic therapy. He was then followed over a 12-month period, with clinical evaluations conducted every 3 months and radiological assessments using a CT scan performed at 6 months and again at 12 months. The patient tolerated the treatment well without complications, recurrence, or metastasis for more than a year of close monitoring.

## 4 Discussion

The World Health Organization (WHO) suggested for the first time in 1972 the term primary intraosseous squamous cell carcinoma (PIOSCC) [5]. In 2005, the WHO Classification of Tumors subcategorized the primary intraosseous squamous cell carcinoma into three groups according to the origin [6]:

Type 1: Solid type, that is considered to be derived from odontogenic epithelial remnants and invade the marrow spaces and induce osseous resorption.
Type 2: Squamous cell carcinoma arising from the lining of an odontogenic cyst, a category that is subdivided into carcinomas arising in keratocystic odontogenic tumors and carcinomas arising in other odontogenic cysts.
Type 3: Squamous cell carcinoma in association with other benign epithelial odontogenic tumors.

The diagnosis of primary intraosseous squamous cell carcinoma is very difficult. Gardner in 1975 proposed the following criteria for the diagnosis of squamous cell carcinoma arising in an odontogenic cyst [7]:

i) It should be confirmed histologically that the epithelial lining of the cyst has undergone malignant transformation to squamous cell carcinoma;
ii) Clinical examination must eliminate gingival squamous cell carcinoma, especially in advanced cases that show direct invasion of cortical bone and fusion to oral mucosa, and the lesion should be located centrally within the bone tissue of the jaws;
iii) No primary neoplasm at distant sites should be detected.

Later Waldron and Mustoe added a fourth criteria: ruling out the possibility of metastatic carcinoma from a distant primary tumor by physical and radiological examinations and the subsequent clinical course [8].

In the current case, the patient had no prior history of squamous cell carcinoma, and the covering gingival mucosa showed normal color despite marked expansive swelling of the mass. Histologically, no morphological features suggested mucosal origin, and the area of transition from benign follicular ameloblastoma to invasive squamous cell carcinoma was clearly evident. Immunohistochemically, the overexpression of p53 and the higher Ki-67 proliferation index, showed the evidence of conspicuous difference between in the squamous cell carcinoma component as compared to the ameloblastoma areas. Therefore, according to Gardner's criteria, this case was diagnosed primary intraosseous squamous cell carcinoma arising from a follicular ameloblastoma, as it fulfilled the criteria detailed below.

The precise molecular mechanism underlying the malignant transformation remains unclear. Nevertheless, the observed overexpression of p53 in the squamous cell carcinoma component suggests that a p53 mutation may have contributed to the process [9].

Hamakawa et al. [10] reported a case demonstrating the histological coexistence of squamous cell carcinoma and mandibular ameloblastoma. In this case, two theories were possible. One is that the SCC developed from the ameloblastoma. The other may be that the ameloblastoma and SCC developed synchronously from remnants of odontogenic epithelium. However, the SCC and the ameloblastoma were observed concurrently and there were no clear histological features of malignant transformation in the report. They suggested that both tumors had occurred synchronously.

On the other hand, Rais and El-Mofty [9] reported a case of desmoplastic ameloblastoma transforming into squamous cell carcinoma. Another case reported by Hiroshi et Al demonstrated the progression of squamous cell carcinoma from a mandibular follicular ameloblastoma [11]. However, in these cases, the evidence of malignant progression was evident radiologically, histologically, and immunohistochemically, which was similar to that seen in our patient.

Those cases illustrate the difficulties that can arise with biphasic lesions with both malignant and benign epithelial components. In our case, the lesion was difficult to diagnose as a malignant tumor based only on intraoral examination and Radiological images. Computed tomography showed an expansive lesion with lytic changes but could not reveal the presence of a malignant area. These factors suggest the primordial role of a histopathological examination before initial treatment.

Early surgical resection followed by adjuvant radiotherapy remains the treatment of choice for avoiding recurrence. The prognosis of primary intraosseous squamous cell carcinoma (PIOSCC) remains uncertain, due to their rare occurrence, but generally carries a poor prognosis. However, cases involving well-differentiated tumors tend to show more favorable outcomes. Histological grading and lymph node metastasis have been identified as key prognostic factors in individual cases [5].

Close monitoring and careful follow-up are essential for the early detection of any signs of recurrence. Its frequency depends on each individual case, based on the patient's overall condition, the progression of the disease and the presence or absence of metastasis.

## 5 Conclusion

Ameloblastoma is considered to be a slow-growing tumor, but its malignant transformation into squamous cell carcinoma can have devastating effects, even if it is very rare. To our knowledge, there were only few cases reporting the coexistence of both tumors in published literature, and the possibility of malignant progression from the benign tumor was not evident in most of them.

## CRediT authorship contribution statement

All authors have read and approved the final manuscript.

**Hanae Ben Abdenbi**: Conceptualization, Methodology, Supervision, Writing- original draft, Writing- review and editing.

**Hafsa El Ouazzani**: Conceptualization, Supervision, Validation, Writing- review and editing.

**Habiba Kadiri**: Methodology, Resources, Supervision.

**Firdaous Touarsa**: Resources, Methodology.

**Nadia Cherradi**: Conceptualization, Supervision.

## Ethical approval

Ethical approval is not applicable. The case report is not containing any personal information. Our institution does not require ethical approval for reporting individual cases or case series.

## Guarantor

Dr. Hanae Ben Abdenbi.

## Funding

No external funding sources are relevant to this submission.

## Patient consent for publication

Written informed consent was obtained from the patient for publication and any accompanying images. A copy of the written consent is available for review by the Editor-in-Chief of this journal on request.

## Declaration of competing interest

The authors declare that they have no competing interests.

## Data Availability

No new data were generated or analyzed in support of this research. The data that support the findings of this study are available from the corresponding author upon reasonable request.
